# Challenges in combating arboviral infections

**DOI:** 10.1038/s41467-024-47161-3

**Published:** 2024-04-18

## Abstract

Arboviral infections are major public health threats, with 100 million people estimated to get sick annually from dengue infection alone. Globally, the risk of arboviruses is likely to further increase both within, and outside of, affected regions due to a combination of factors including climate change, human mobility, and other societal factors. Despite the availability of vaccines for some arbovirus infections, there is a lack of specific antiviral treatment options. *Professor Johan Neyts* at the University of Leuven, Belgium, has been working on developing antiviral strategies for more than 30 years. His current research focuses on developing antiviral drugs and vaccines against emerging and neglected viruses many of which are arboviruses. In this Q&A, he discusses the risks associated with vector-borne virus infections, challenges in developing efficient drugs for treatment, and current promising efforts to address these challenges.

**Figure:**
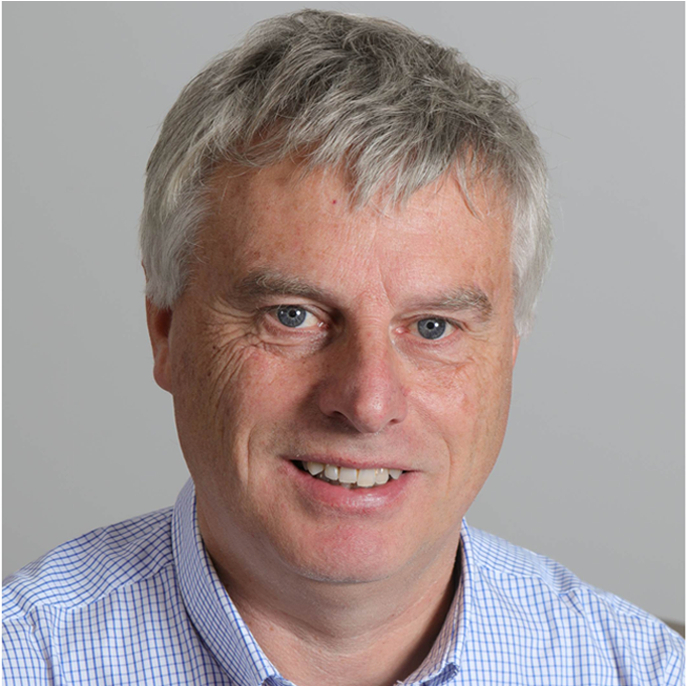
Dr. Johan Neyts, Professor at the University of Leuven, Belgium.

**1. What are arboviruses and why are they such a public health concern?**

Arboviruses (arthropod-borne viruses) infect people and animals through the bite of infected vectors. They are typically either flaviviruses, alphaviruses, or bunyaviruses; examples of arboviral diseases include yellow fever, dengue, West Nile, Zika, Chikungunya, and Rift Valley fever (which are all transmitted by mosquitoes), and tick-borne encephalitis, and Crimean-Congo hemorrhagic fever (which are all transmitted by ticks). Sandflies can also be a vector for some bunyaviruses. Arboviruses can also be transmitted by blood transfusion, organ transplantation, sexual contact, and through the placenta during pregnancy. Arboviral-associated diseases have a wide range of mild to severe symptoms including febrile illnesses, encephalitis, and hemorrhagic fevers.

They are major public health threats in tropical and subtropical regions, where almost 4 billion people live, and they are often difficult to control. Part of the complications in public health management of arboviral-associated diseases is that current treatment is solely based on the management of symptoms. For example, in the case of dengue, which is now endemic in more than 100 countries, the situation is complex as the standard treatment for severe disease is fluid resuscitation to manage the common symptoms of plasma leakage and organ hypoperfusion while the infection runs its course, and access to care is often limited in high-risk, rural areas.

The significance of the public health burden of arboviruses led to the 2022 launch of the Global Arbovirus Initiative by the WHO. During the launch event Dr. Sylvie Briand, the WHO Director of Pandemic and Epidemic Diseases, said that the next pandemic could be due to a new arbovirus and that there are already indications that the risk is increasing. The new WHO initiative provides a list of priority actions that countries and regions can implement in preparation for future arbovirus outbreaks, which include facilitating global-scale real-time surveillance and strengthening efforts to develop new diagnostics, drugs, and vaccines. The Global Arbovirus Initiative is complementary to other WHO initiatives, namely, the Neglected Tropical Disease Roadmap, the Global Vector Control Response Initiative, and the Eliminate Yellow Fever Epidemics (EYE) strategy. The latter aims to stop yellow fever, which is responsible for ~200,000 annual cases and ~30,000 deaths, mostly in Africa, by 2026.

**2. There are a number of preventive measures available for arboviruses, which mostly include vaccines. Additionally, a range of vector control measures are implemented in endemic regions. Why do we still need to think about treatment options for these diseases?**

When talking about vaccines as preventive measures against arboviruses, the yellow fever vaccine, which has been in use since 1938, deserves to be highlighted. It is a live-attenuated virus and a single dose provides protection for 80–99% of people within 10 days of vaccination; 99% of people have life-long protection against yellow fever disease within 30 days of vaccination, which makes the vaccine one of the most efficacious for any infectious disease. Yet, many people still do not get vaccinated. For example, in the African region routine immunization coverage against yellow fever for childhood vaccinations was only 48%, which is much lower than the threshold of 80% needed for population immunity against the virus (www.who.int). The vaccine needs to be stored and transported under strict cold-chain conditions, which is often complicated in remote tropical regions.

The first dengue vaccine (Dengvaxia®, Sanofi) was approved for use in patients aged 9 years and above in Mexico, the Philippines, and Brazil in 2015 and in El Salvador, Costa Rica, Paraguay, Guatemala, Peru, Indonesia, Thailand, and Singapore in 2016; it was also approved for use in Europe in 2018. A second dengue vaccine (QDENGA®, Takeda) was approved for use in Europe, Brazil, Argentina, Indonesia, and Thailand in 2022. Dengue vaccine development has proven to be challenging because of the existence of four virus serotypes. Dengue vaccines should be tetravalent and must induce a balanced immune response so that the antibody levels against each of the serotypes are sufficiently high. This is to avoid a phenomenon called antibody-dependent enhancement (ADE). Previous exposure to dengue may, in a delicate balance and as soon as cross-protective antibodies drop below a certain critical threshold, increase the risk of more severe disease when the patient is infected with another serotype. Although the mechanism of ADE is not yet fully understood, it is thought to result from a more efficient uptake of the virus by monocytes and macrophages which is facilitated by suboptimal levels of non-neutralizing DENV-specific antibodies. In vaccine efficacy studies, individuals who were vaccinated and then acquired a natural dengue infection had a somewhat higher risk of severe disease due to ADE; as a consequence, the WHO advises pre-vaccination screening be conducted, and to only vaccinate individuals who have evidence of a previous natural dengue infection. This limits the total number of individuals who can be vaccinated in dengue-endemic countries, which impacts control/preventive measures. Vaccines are available against a limited number of other arboviruses, such as the Japanese encephalitis virus (JEV) and the tick-borne encephalitis virus (TBEV). More recently the first vaccine has been licensed against the chikungunya virus. Overall, and of course not limited to vaccines against arboviruses, there are a number of challenges when implementing vaccination strategies against certain diseases. Some vaccines might, for example, be contra-indicated for some groups (such as the use of live-attenuated vaccines in pregnant women or immunodeficient patients). The need for a strict cold-chain may make it difficult to bring vaccines to populations in remote rural tropical regions. Also, vaccine hesitancy may be a factor resulting in suboptimal vaccine coverage.

From a preventive perspective, vector control measures remain critical. The most widely used approaches are the use of larvicides (to kill mosquito larvae) by direct application in stagnant water or spraying insecticides to kill adult mosquitoes. An interesting experimental approach is the release of *Aedes aegypti* mosquitoes with a reduced ability to transmit viruses to humans (which is achieved by infecting them with *Wolbachia* bacteria). Breeding of such infected mosquitoes with the wild mosquito population ultimately reduces the number of mosquitoes that are efficient vectors. This has, for example, been implemented in a controlled trial in Yogyakarta, Indonesia^1^. Another approach is releasing billions of sterile male mosquitoes to mate with females in the wild. This Sterile Insect Technique (SIT) is being tested in Tahiti where the impact of the technology on dengue transmission will be measured for the first time (www.who.int). While these measures may be effective in reducing local mosquito populations in some areas, achieving complete vector control in all affected regions globally seems to be a Sisyphus job as they are very localized or are still at an experimental stage.

Therefore, despite available vector control measures and vaccines against multiple arboviral diseases, a large number of people still become infected and may develop severe diseases. It will be important to have antiviral drugs at hand both for prophylaxis and treatment. In the case of dengue, for example, there is a strong case for developing antiviral prophylactics where the aim may also be to reduce household and community transmission during outbreaks. Also, travelers to dengue-endemic regions may benefit from such prophylaxis. The strategy may somehow be compared to the prophylaxis against malaria. In the context of oral antiviral treatment, one may expect treated dengue patients to have lower plasma viremia levels. This may also have a beneficial effect on transmission of the virus; since mosquitoes will ingest a lower virus inoculum during blood meals making them less efficient viral vectors. Additionally, there is evidence that mosquitoes will also consume antiviral drugs during the blood meal and studies have shown that in such cases antiviral molecules block viral amplification in the mosquito, which directly affects transmission.

**3. What are the challenges for developing effective antiviral treatments for infections with flaviviruses in general, and dengue virus in particular?**

In principle, it is possible to develop antivirals that act against an entire genus or family of viruses and that directly act on viral targets, also known as direct-acting antivirals (DAA). Highly potent and safe antiviral drugs have been developed against a number of viral infections and there is no reason to believe that this would not be possible against flaviviruses and other (arbo)viruses. The best showcase for the power that antiviral drugs may have is their successful use in the treatment of infections with HIV, HBV, or HCV. Today chronic HBV, HCV, and HIV infections can be effectively controlled with just one or two daily pill(s) that consist, in the case of HIV and HCV, of a combination of multiple drugs with non-overlapping resistance profiles. There are now even long-acting formulations available that allow effective treatment of HIV infections with one injection every couple of months.

An important difference between infections with HIV, HBV, and HCV, and flavivirus infections is that the latter cause acute infections; consequently, the treatment initiation window for flaviviruses is typically short. For example, in the case of dengue, because of the short duration of viremia, treatment will need to be initiated as soon as possible following symptom onset. Additionally, although flaviviruses and HCV belong to the same family (Flaviviridae), there are important differences in their genomic organization and replication biology. For example, the NS5A protein of HCV is an important drug-target, but it has no homolog in flaviviruses. Also, although HCV and flaviviruses have related proteases, the HCV protease inhibitors do not inhibit the replication of flaviviruses, because the enzymes are too divergent. There are also major genetic differences between flaviviruses; for example, the dengue, yellow fever, and Japanese encephalitis viruses have significant genetic differences. In the case of dengue, there are four fairly divergent serotypes that could almost be considered four different viruses. This means that some potential drug targets may structurally vary so much that it may be difficult for an inhibitor to be equipotent against all serotypes, despite the need for antiviral drugs against dengue to exert equipotent pan-serotype activity.

**4. You, and your co-workers from various disciplines, have developed a drug that effectively works against all four serotypes of the dengue virus. It also shows promising safety profiles in animals and in humans. What is the story behind this compound and what makes it so effective and yet safe?**

Almost 20 years ago, my laboratory started working on the development of antiviral drugs and strategies against HCV. Together with the Swiss company DebioPharm, we discovered Alisporivir as, a potent inhibitor of HCV and deciphered its mechanism of antiviral activity. Alisporivir made it to Phase 3 clinical studies. Together with a team at the University of Innsbruck in Austria, we also developed a class of potent non-nucleoside HCV polymerase inhibitors which Gilead Sciences advanced to Phase 2 clinical studies. These were exciting times that inspired me to try to also develop highly efficacious drugs against dengue viruses, which, as said, belong to the same family as HCV.

We started our efforts together with the Center for Drug Design and Development (www.cd3.be) and funded by the Wellcome Trust, by screening a large library of small molecules with drug-like properties in a phenotypic antiviral assay [a dengue virus type 2 (DENV2) infection assay in Vero cells] in the hope to identify molecules that resulted in complete inhibition of viral replication without an adverse effect on the host cells. A 3-acyl-indole was identified as such a “hit”. A molecule identified in a screen is obviously not yet a drug candidate and the potency and drug-like properties of such hits need to be improved. In this particular case, there was an extra level of complexity; the hit identified against DENV2 proved to be 10- to 50-fold less active against the other dengue serotypes. Various analogs of the hit molecule were synthesized; some were equipotent, some had lost all antiviral activity, and some were more potent than the hit identified on the screen. This information guided the medicinal chemists in the team, to understand which chemical modifications were needed to improve the potency against each of the four serotypes. In a stepwise approach and after almost 2000 analogs had been synthesized, pan-serotype inhibitors active at picomolar concentrations were identified^2,3^. Because each of the four serotypes of dengue also have different genotypes, the team of Xavier de Lamballerie at the University of Aix-Marseille tested the antiviral molecules against all 21 genotypes of DENV; all of these strains were highly susceptible to the inhibitor^4^.

In addition to the antiviral potency and selectivity (which is a potent antiviral effect without adverse effects on the host cell) the physicochemical properties, pharmacokinetic, and toxicological profiles were optimized. To further advance this class of molecules toward clinical development, the expertise of a pharmaceutical company experienced in antiviral drug development was needed. We were pleased that Janssen Pharmaceutic (J&J) Global Public Health joined us in our efforts. Finally, in a joint effort, potent pan-serotype inhibitors were obtained that resulted in excellent antiviral activity upon oral dosing in mouse and nonhuman primate infection models of dengue^4,5^. Resistance development in cell culture appeared to be a slow (~3–6 months) process and 3–4 mutations (in the viral NS4b gene) are needed to confer full resistance. Together, these mutations do not pre-exist in the wild-type virus population. Interestingly, the drug-resistant variants against this antiviral series do not replicate in mosquito cells^4^. It may, thus, be assumed that even if drug-resistant variants would emerge in some patients, such strains may not replicate to sufficiently high titers in mosquitoes to enable transmission. Since the class of inhibitors was originally identified through a phenotypic screening, the molecular mechanism by which they block the replication of the virus was still unclear. To solve that question, we were joined by the team of Ralf Bartenschlager at the University of Heidelberg, who demonstrated that the molecules prevent an interaction between two viral proteins (in fact between NS2B/NS3 and NS4A-2K-NS4B) that is essential for viral replication to proceed. These viral proteins have no human homologs, the molecular target is thus virus-specific, which results in a safe and tolerable profile in animals.

One molecule from this chemical series (with code name JNJ-1802) is now in clinical trials. At the end of October 2023, J&J presented promising data from a Phase 2a human DENV-3 challenge study with JNJ-1802, at the annual meeting of the American Society of Tropical Medicine & Hygiene in Chicago. The molecule was reported to be safe and well-tolerated. Healthy participants received daily doses of JNJ-1802, or a placebo, for 26 days and were infected on the 5th day with the virus. Six of the ten participants who received the highest evaluated dose of JNJ-1802 had no detectable virus in their blood throughout the study, whereas all of the placebo recipients had detectable levels of virus^6^.

**5. How well do you think small molecule inhibitors, such as the one you have developed for dengue, can be implemented in endemic areas with limited resources? Are there important considerations for treatment in low and middle-income countries (LMICs)?**

An important advantage of the use of oral antivirals is their potential rollout as antiviral prophylaxis for the control of outbreaks, which is likely to be faster than for vaccines as they typically do not require the strict cold chain that vaccines and antibody therapies require. The supply-chain logistics and the so-called last-mile, which refers to the final factors that complicate the transportation of vaccines to their final destination including temperature or mode of transportation, might be less complicated than is the case for vaccines. In fact, the supply-chain logistics may be more or less comparable to the situation with anti-HIV drugs that also need to reach patients in LMICs who often live in remote tropical regions, with less efficient infrastructures and means of transportation. In addition, such drugs exert their pharmacological effect within hours whereas in the case of many vaccines, there is often an immunity gap that may last one to several weeks.

**6. Do you think that it is possible that we will eventually move to universal flavivirus treatments, or those that can be used to target more than one disease?**

It is likely that it will not be economically viable to develop antiviral drugs against every individual flavivirus that is pathogenic to humans, which reinforces the need for pan-flavivirus inhibitors. Such molecules are not only needed for the treatment of infections with known flaviviruses but they will also be needed for epidemic and pandemic preparedness. The 2014–2015 Zika virus outbreak demonstrated how rapidly this flavivirus was able to spread in immunologically naïve Latin-American populations; it highlighted the importance of having stockpiles of potent pan-flavivirus inhibitors available in the event of outbreaks with new and highly pathogenic flaviviruses. There are druggable targets in the replication cycle of flaviviruses that may be explored for the development of pan-flavivirus drugs, for example, I believe that viral enzymes such as the NS3 viral protease and the RNA-dependent RNA polymerase, are excellent targets to that end. Nucleoside analogs that inhibit the replication of a broad panel of flaviviruses by targeting a crucial viral enzyme have already been reported (such as NITD-008 and 2′-C-methylcytidine), although these have, for various reasons, not been further developed. One important consideration is that flaviviruses can vary in their resulting infections; dengue and yellow fever, for instance, cause systemic infections, whereas other flaviviruses are neuroinvasive/neurotropic. It remains to be seen if there is a case for developing two separate groups of pan-flavivirus inhibitors of which one is optimized to result in high exposure of the drug in the central nervous system.

*This interview was conducted by Dr. Danielle Troppens*.
